# Autologous or heterologous fibrin sealant scaffold: which is the better choice?

**DOI:** 10.1186/1678-9199-20-31

**Published:** 2014-07-18

**Authors:** Rui Seabra Ferreira

**Affiliations:** 1 Center for the Study of Venoms and Venomous Animals (CEVAP), São Paulo State University (UNESP – Univ Estadual Paulista), Botucatu, São Paulo State, Brazil, Rua José Barbosa de Barros, 1780, Fazenda Experimental Lageado, Botucatu, SP CEP 18.610-307, Brasil

**Keywords:** Fibrin sealant, Snake venom, Scaffold, Stem cell

## Dear Editor-in-chief

Since the foundation of the *Journal of Venomous Animals and Toxins including Tropical Diseases* in 1995, special attention has been given to the publication of results from research studies on products developed from animal toxins [1,2]. For years, proteins, enzymes and peptides have been isolated and synthesized in the search for new medications and target molecules. The serine proteases extracted from snake venom were selected as molecules of interest since they act on human and animal hemostatic systems, by the thrombin-like enzymes, converting fibrinogen into fibrin [1,2].

About 20 years ago the researchers of the Center for the Study of Venoms and Venomous Animals (CEVAP) at UNESP proposed to engage in prospecting for a new sealant from a serine protease extracted from the venom of *Crotalus durissus terrificus* associated with a fibrinogen-rich cryoprecipitate and coagulation factors extracted from buffalos [1,3,4]. For medicinal application, this new sealant does not present risk of transmission of infectious diseases, is cheaper on account of being a genuinely Brazilian product, is standardized and was already tested experimentally on rodents (rats and rabbits) and non-rodents (dogs and sheep) [2-5]. In humans, the fibrin sealant was tested in a autologous skin graft of the nasogenian sulcus as donor site to repair a nasal surgical wound, in the immobilization of free periodontal gingival grafts in lower premolars, in the fixation of a conjunctive tissue graft for the correction of gingival marginal tissue recessions and in the treatment of chronic venous ulcers [6-10].

Recently, we have investigated the effects of the fibrin sealant as a scaffold for mesenchymal stem cells in order to demonstrate the ability of cells to interact with the biological microenvironment [11]. We observed that the product did not affect cell adhesion, proliferation or differentiation and allowed the adherence and growth of mesenchymal stem cells on its surface. Hoechst 33342 and propidium iodide staining demonstrated the viability of mesenchymal stem cells in contact with the fibrin sealant and the ability of the biomaterial to maintain cell survival. We concluded that the new biomaterial is a three-dimensional scaffolding candidate that is capable of maintaining cell survival without interfering with differentiation, and might also be useful in drug delivery. The fibrin sealant has a low production cost, does not transmit infectious diseases from human blood and has properties of a suitable scaffold for stem cells because it permits the preparation of differentiated scaffolds that are suitable for every need.

Similarly, de la Puente *et al.*[12-14] developed an autologous fibrin scaffold: cheaper, easy to use, natural (with physiological concentrations of fibrinogen), implantable, highly available and with low fibrinogen concentrations. The key point to this model is that no fibrinogen concentration techniques, such as cryoprecipitation or other chemical methods, are used. This system is suitable both for cell culture and cell differentiation. Autologous blood is collected, centrifuged to separate it from the plasma (physiological concentrations in plasma are 2–4 mg/mL) and frozen at -40°C until used. Accordingly, more natural and fewer modified fibrins are used and the nature of the fibrin (with its physiological concentrations) is preserved. The authors concluded that the structuring and development of the scaffold is a key point for cell culture because scaffolds of autologous fibrin offer an important alternative due to their low fibrinogen concentrations, which are more suitable for cell growth [12-14].

The results of de la Puente *et al.*[12-14] are in disagreement with those of Gasparotto *et al.*[11], since the scaffold of the latter is of animal origin (constituted by fibrinogen extracted from buffalos through the cryoprecipitation technique), contains elevated fibrinogen concentrations, permits good growth of cells in a new biomaterial, and poses no risk of transmitting infectious diseases by means of human blood. Although both sealants (autologous and heterologous) are natural, biodegradable, bio-absorbent, non-toxic and non-immunogenic, future comparative studies using human stem cells should be carried out to clarify these points.

## Competing interests

The author declares that there are no competing interests.
